# Supplementary material for: Pyruvate Dehydrogenase Kinase 1 inhibition mediated oxidative phosphorylation enhancement in cartilage promotes osteoarthritis progression
Source: BMC Musculoskelet Disord. 2023 Jul 20;24:597. doi: 10.1186/s12891-023-06585-6 (PMC10357736; doi:10.1186/s12891-023-06585-6)
Supplement: Supplementary file 1 — Additional file 1. [file 12891_2023_6585_MOESM1_ESM.zip › KEGG.Gsea.1653622432464/KEGG_GLYCINE_SERINE_AND_THREONINE_METABOLISM.html]

Details for gene set KEGG\_GLYCINE\_SERINE\_AND\_THREONINE\_METABOLISM[GSEA]

|  || Dataset | 55457\_collapsed\_to\_symbols.cls.cls #OA\_versus\_normal.cls.cls #OA\_versus\_normal\_repos |
| Phenotype | cls.cls#OA\_versus\_normal\_repos |
| Upregulated in class | OA |
| GeneSet | KEGG\_GLYCINE\_SERINE\_AND\_THREONINE\_METABOLISM |
| Enrichment Score (ES) | 0.32832614 |
| Normalized Enrichment Score (NES) | 1.1292027 |
| Nominal p-value | 0.27033493 |
| FDR q-value | 0.44478327 |
| FWER p-Value | 1.0 |
Table: GSEA Results Summary

  

Fig 1: Enrichment plot: KEGG\_GLYCINE\_SERINE\_AND\_THREONINE\_METABOLISM      
 Profile of the Running ES Score & Positions of GeneSet Members on the Rank Ordered List

  

| SYMBOL | TITLE | RANK IN GENE LIST | RANK METRIC SCORE | RUNNING ES | CORE ENRICHMENT || 1 | GATM | glycine amidinotransferase [Source:HGNC Symbol;Acc:HGNC:4175] | 56 | 0.976 | 0.1123 | Yes |
| 2 | ALAS1 | 5'-aminolevulinate synthase 1 [Source:HGNC Symbol;Acc:HGNC:396] | 288 | 0.757 | 0.1848 | Yes |
| 3 | DLD | dihydrolipoamide dehydrogenase [Source:HGNC Symbol;Acc:HGNC:2898] | 565 | 0.635 | 0.2391 | Yes |
| 4 | GCAT | glycine C-acetyltransferase [Source:HGNC Symbol;Acc:HGNC:4188] | 1095 | 0.497 | 0.2575 | Yes |
| 5 | GAMT | guanidinoacetate N-methyltransferase [Source:HGNC Symbol;Acc:HGNC:4136] | 1418 | 0.441 | 0.2851 | Yes |
| 6 | SHMT2 | serine hydroxymethyltransferase 2 [Source:HGNC Symbol;Acc:HGNC:10852] | 1518 | 0.426 | 0.3283 | Yes |
| 7 | MAOB | monoamine oxidase B [Source:HGNC Symbol;Acc:HGNC:6834] | 2192 | 0.339 | 0.3166 | No |
| 8 | GLDC | glycine decarboxylase [Source:HGNC Symbol;Acc:HGNC:4313] | 3913 | 0.163 | 0.2025 | No |
| 9 | SRR | serine racemase [Source:HGNC Symbol;Acc:HGNC:14398] | 4397 | 0.121 | 0.1794 | No |
| 10 | SDS | serine dehydratase [Source:HGNC Symbol;Acc:HGNC:10691] | 4579 | 0.106 | 0.1781 | No |
| 11 | CBS | cystathionine beta-synthase [Source:HGNC Symbol;Acc:HGNC:1550] | 5285 | 0.049 | 0.1291 | No |
| 12 | DAO | D-amino acid oxidase [Source:HGNC Symbol;Acc:HGNC:2671] | 6252 | -0.024 | 0.0570 | No |
| 13 | PSAT1 | phosphoserine aminotransferase 1 [Source:HGNC Symbol;Acc:HGNC:19129] | 6585 | -0.049 | 0.0370 | No |
| 14 | PIPOX | pipecolic acid and sarcosine oxidase [Source:HGNC Symbol;Acc:HGNC:17804] | 6781 | -0.061 | 0.0292 | No |
| 15 | SARDH | sarcosine dehydrogenase [Source:HGNC Symbol;Acc:HGNC:10536] | 7211 | -0.097 | 0.0074 | No |
| 16 | BHMT | betaine--homocysteine S-methyltransferase [Source:HGNC Symbol;Acc:HGNC:1047] | 7250 | -0.099 | 0.0163 | No |
| 17 | AGXT | alanine--glyoxylate and serine--pyruvate aminotransferase [Source:HGNC Symbol;Acc:HGNC:341] | 8115 | -0.164 | -0.0312 | No |
| 18 | MAOA | monoamine oxidase A [Source:HGNC Symbol;Acc:HGNC:6833] | 8271 | -0.177 | -0.0221 | No |
| 19 | AMT | aminomethyltransferase [Source:HGNC Symbol;Acc:HGNC:473] | 9021 | -0.234 | -0.0524 | No |
| 20 | GNMT | glycine N-methyltransferase [Source:HGNC Symbol;Acc:HGNC:4415] | 9208 | -0.248 | -0.0372 | No |
| 21 | PHGDH | phosphoglycerate dehydrogenase [Source:HGNC Symbol;Acc:HGNC:8923] | 9245 | -0.252 | -0.0099 | No |
| 22 | AOC3 | amine oxidase copper containing 3 [Source:HGNC Symbol;Acc:HGNC:550] | 9375 | -0.262 | 0.0113 | No |
| 23 | SHMT1 | serine hydroxymethyltransferase 1 [Source:HGNC Symbol;Acc:HGNC:10850] | 9727 | -0.294 | 0.0192 | No |
| 24 | ALAS2 | 5'-aminolevulinate synthase 2 [Source:HGNC Symbol;Acc:HGNC:397] | 10247 | -0.346 | 0.0202 | No |
| 25 | CTH | cystathionine gamma-lyase [Source:HGNC Symbol;Acc:HGNC:2501] | 11108 | -0.444 | 0.0065 | No |
| 26 | AOC2 | amine oxidase copper containing 2 [Source:HGNC Symbol;Acc:HGNC:549] | 11550 | -0.508 | 0.0329 | No |
| 27 | PSPH | phosphoserine phosphatase [Source:HGNC Symbol;Acc:HGNC:9577] | 12036 | -0.602 | 0.0671 | No |
Table: GSEA details [plain text format]

  

Fig 2: KEGG\_GLYCINE\_SERINE\_AND\_THREONINE\_METABOLISM      
 Blue-Pink O' Gram in the Space of the Analyzed GeneSet

  

Fig 3: KEGG\_GLYCINE\_SERINE\_AND\_THREONINE\_METABOLISM: Random ES distribution      
 Gene set null distribution of ES for **KEGG\_GLYCINE\_SERINE\_AND\_THREONINE\_METABOLISM**
